# Nonlinear Gait Variability and the Role of Cognitive-Physical Exercise in Mitigating Mobility Decline in Institutionalized Older Adults with Cognitive Impairment

**DOI:** 10.3390/jfmk11010097

**Published:** 2026-02-26

**Authors:** João Galrinho, Marco Batista, Marta Gonçalves-Montera, Ana Rita Matias, Orlando Fernandes

**Affiliations:** 1 Comprehensive Health Research Center (CHRC), Department of Sports and Health, School of Health and Human Development, University of Evora, 7006-071 Évora, Portugal; armatias@uevora.pt; 2 Sport Physical Activity and Health Research & Innovation Centre, SPRINT Polytechnic University of Castelo Branco, 6000-084 Castelo Branco, Portugal; marco.batista@ipcb.pt; 3 Faculty of Psychology, University of Lisbon, 1649-013 Lisbon, Portugal; martagoncalves@psicologia.ulisboa.pt

**Keywords:** gait variability, Sample Entropy, dual-task, cognitive impairment, exercise, aging

## Abstract

**Background:** Age-related cognitive decline is linked to reduced gait complexity and higher fall risk. Traditional linear gait measures may miss subtle motor-cognitive deficits in older adults with dementia. This study examined whether an 8-week motor-cognitive exercise program could improve gait adaptability in institutionalized older adults with cognitive impairment. Gait complexity, measured using Sample Entropy, was the primary outcome. **Methods:** Forty-two institutionalized older adults completed follow-up assessments, including 26 with cognitive impairment and 16 controls. Gait was assessed during normal walking (single-task) and while performing cognitive tasks (dual-task), such as naming animals or counting backward. Inertial sensors recorded stride intervals, and Sample Entropy was calculated to evaluate gait regularity and adaptability, (gait complexity). The intervention included 24 structured sessions combining physical and cognitive exercises targeting balance, coordination, and executive function. Non-parametric tests (Wilcoxon) were used, with Bonferroni correction for multiple comparisons. **Results:** Participants with cognitive impairment showed increased gait complexity, especially during dual-task walking. Significant improvements were found in both limbs under dual-task conditions (left: *p* = 0.015, effect size = 0.34; right: *p* = 0.030, effect size = 0.31). During single-task walking, a significant improvement was observed in the left limb (*p* = 0.006, effect size = 0.39). **Conclusions:** Motor-cognitive exercise may enhance non-linear gait complexity in institutionalized older adults with cognitive impairment. The use of dual-task training in rehabilitation and highlight the value of entropy-based gait assessment for detecting subtle functional changes. However, the lack of a randomized non-exercising cognitive impairment control group limits definitive conclusions about causality.

## 1. Introduction

Demographic aging is one of the biggest public health challenges of the 21st century. It affects healthcare systems, as well as economic and social structures. According to the World Health Organization [1], the number of people aged 60 years or older is increasing quickly. By 2050, this group is expected to represent more than 20% of the world’s population. As people age, they are more likely to develop chronic diseases, frailty, and cognitive problems, such as dementia and Mild Cognitive Impairment. In institutionalized older adults, cognitive impairment is linked to a higher risk of losing independence, needing care, and early death [2].

One clear effect of cognitive decline is reduced mobility. Simple tasks, such as standing up from a chair or walking, can become more difficult. This greatly increases the risk of falls, which are the main cause of injury, hospitalization, and accidental death in older adults [3]. Reduced mobility also harms mental health and social interaction, often leading to isolation and greater dependence. Early assessment of mobility in institutionalized older adults with cognitive impairment is very important for timely intervention. Two commonly used tools are the Timed Up and Go Test, a quick and practical way to assess mobility and fall risk [4], and the 10-Meter Walk Test, which measures walking speed and quality—important indicators of independence and life expectancy [5].

In recent years, there has been growing interest in assessing mobility under dual-task conditions. In these situations, a person performs a physical task while also doing a cognitive task, such as counting backwards or naming animals. These tasks reflect real-life situations where attention must be divided. Studies show that dual-tasking slows walking speed and increases the time needed to complete tasks, especially in people with cognitive impairment. This shows the strong link between cognitive and motor functions [3,6].

Recent research challenges the idea that walking is only a simple and automatic motor task. Evidence shows that walking, especially in complex environments, depends on higher brain functions, such as attention and working memory [7,8]. This is explained by the Cognitive-Motor Interference theory. This theory suggests that when a person performs a motor and a cognitive task at the same time, both tasks compete for limited brain resources, leading to worse performance in one or both tasks [9].

In addition, aging of the neuromuscular system creates extra challenges. The age-related loss of muscle mass and function, known as sarcopenia, changes how muscles are activated and reduces movement efficiency [10,11]. These changes, combined with cognitive decline, increase postural instability and make the brain work harder to control walking [12]. Because of this, dual-task walking assessment is not only a physical test, but also a way to understand how brain networks are working [5,13]. Cognitive impairment affects both thinking and movement, leading to abnormal walking patterns and a higher risk of falls [5]. However, traditional linear measures of gait often fail to capture the complexity of these changes [14]. In this study, gait complexity is defined as the pattern and regularity of steps over time, reflecting the ability of the neuromotor system to adapt.

Exercise programs designed for older adults have shown good results in improving both cognitive and motor abilities. Programs that include strength training, balance exercises, mobility exercises, and cognitive tasks can improve physical performance, reduce the risk of falls, and may slow cognitive decline [15,16]. However, more research is needed to understand how these programs affect institutionalized older adults with cognitive impairment, especially in dual-task situations.

This study aims to evaluate the effects of an 8-week motor-cognitive exercise program on the functional mobility of institutionalized older adults with cognitive impairment. While the 10-Meter Walk Test provides functional information, the main outcome is gait complexity. This is measured using Sample Entropy, which evaluates step-to-step variability in a non-linear way. Combining physical and cognitive tasks may improve the connection between thinking and movement, and enhance walking ability [17,18]. This study uses wearable sensors to examine whether these combined interventions can improve gait patterns.

Finally, this study aims to show that structured motor-cognitive exercise improves the ability to adapt walking patterns. The research hypothesis suggests that the intervention leads to consistent improvements in gait complexity, especially when a cognitive task is involved. This indicates that non-linear measures are important for detecting small but meaningful changes in neuromotor function in this population.

## 2. Materials and Methods

**Figure 1:**
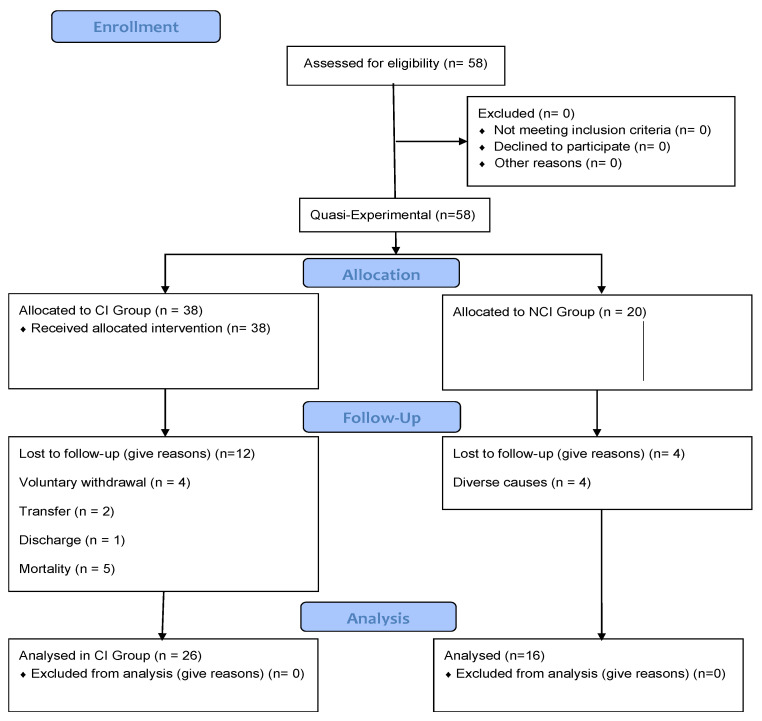
Participant Flow Diagram.

The low chart diagram below illustrates the progression of participants through the study, including recruitment, group allocation, and reasons for attrition (Figure 1). It is presented to enhance transparency and to address potential concerns regarding survival bias.

### 2.1. Ethics Approval

The Évora University research ethics committee approved all procedures (GD 24725/2023). Participants provided written informed consent in accordance with the Helsinki Declaration before participating. All participants also provided verbal consent.

### 2.2. Study Participants

To address potential survival bias, baseline characteristics were compared between participants who completed the intervention and those who did not. No statistically significant differences were observed in age, MMSE score, or baseline Sample Entropy values (*p* > 0.05), suggesting that attrition did not systematically bias the baseline profile of the analyzed sample.

Participants were included in the cognitive impairment group based on Mini-Mental State Examination (MMSE) score cutoffs: 15 or lower for individuals who were illiterate, 22 or lower for those with 1 to 11 years of education, and 27 or lower for those with more than 11 years of education [19,20]. A Clock Drawing Test score of 6 or lower was also required [21,22].

### 2.3. Gait Assessment and Data Processing

Gait was measured using compact Inertial Measurement Units placed just above the malleoli. Participants completed three 10 m walking trials under single-task and dual-task conditions. During the dual-task condition, participants named animals or counted backward while walking to assess cognitive-motor interference [3,6].

Acceleration data were collected at 50 Hz. Sample Entropy (SaEn) was used to measure gait complexity. For reproducibility, the parameters were set as follows m = 2, tolerance r = 0.2 times the standard deviation These parameter values provide a good balance between sensitivity and reliability, work well with short and noisy biological signals, and capture more structure in the signal [23,24]. The time series length N was defined as the total number of strides per trial.

In the single-task condition, the Cognitive Impairment group had an average of 28 ± 5 inter-stride intervals per participant, while the Non-Cognitive Impairment group had 26 ± 4. In the dual-task condition, the averages were 30 ± 6 and 27 ± 5, respectively. Across all groups and conditions, the mean series length was about 28 strides, which is sufficient for stable SaEn estimation in this clinical context.

The data were filtered using a 7th-order Butterworth low-pass filter with a 13 Hz cutoff. The inter-stride intervals (ISIs), defined as the time between consecutive heel strikes of the same foot [25,26], were processed using MATLAB R2022a (MathWorks, Natick, MA, USA).

### 2.4. Intervention

The Mind in Motion (M2) intervention was a multicomponent program based on evidence showing that this type of approach is more effective than single-modality training [27,28]. The program combined balance, gait, and cognitive exercises to promote neuroplasticity [17]. It lasted eight weeks, with 24 sessions of 30 min each [15]. Program fidelity was monitored using session logs and instructor checklists to ensure that the protocol was followed correctly [29].

The program was divided into two phases. Weeks 1 to 4 focused on basic mobility, and weeks 5 to 8 focused on dual-task training that included working memory and auditory response exercises. Each session included a 5 min warm-up, a 20 min main phase, and a 5 min cool-down, following the training principles of the American College of Sports Medicine [30].

### 2.5. Statistical Analysis

Analysis was performed using IBM SPSS (version 30.0). Due to non-normal distribution (Shapiro–Wilk test), non-parametric tests were used: Mann–Whitney U for between-group differences and Wilcoxon Signed-Rank for within-group changes. A Bonferroni correction was applied to all comparisons between limbs and tasks to mitigate Type I errors.

Effect sizes were calculated using Rosenthal’s r formula (r = Z divided by the square root of N) [31]. Magnitude was interpreted as small (0.10 to 0.29), moderate (0.30 to 0.49), or large (above or equal to 0.50) [32]. Significance was set at *p* < 0.05.

### 2.6. Data Availability Statement

The datasets are not readily available as they are part of an ongoing study. Requests should be directed to the corresponding author.

## 3. Results

This section presents the results of the Sample Entropy analysis based on accelerometer data from the left and right lower limbs during single-task and dual-task walking conditions. The analysis compares pre- and post-intervention measurements between the Cognitive Impairment (CI) group and the cognitively healthy Control group (Non-Cognitive Impairment [NCI]). Both statistical significance and effect size were evaluated using Rosenthal’s r.

### 3.1. Statistical Analysis of Gait Entropy

Non-parametric tests indicated that, at baseline, the CI group exhibited lower entropy in the right lower limb compared to the NCI group, with a *p*-value of 0.036 and a moderate effect size of 0.33. In the left limb, the difference at baseline was not statistically significant, with a *p*-value of 0.146 and a small effect size of 0.23. These findings align with reports of reduced gait complexity in individuals with cognitive decline (Table 1).

Within-group comparisons following the intervention revealed distinct trajectories. The CI group exhibited a statistically significant increase in Sample Entropy in the left lower limb, with a *p*-value of 0.006 supported by a moderate effect size of 0.39, indicating an improvement in gait complexity. Regarding the right limb in the CI group, the change did not reach statistical significance, with a *p*-value of 0.220 and a small effect size of 0.17. This limited effect in the right limb under single-task conditions suggests a positive trend that may require more power or a longer duration to confirm. The NCI group remained stable across both limbs, with *p*-values greater than 0.05 and negligible to small effect sizes below 0.20.

Under dual-task conditions, the intervention produced significant increases in Sample Entropy for the CI group in both the left limb, with a *p*-value of 0.015, and the right limb, with a *p*-value of 0.030. These improvements were characterized by moderate effect sizes of 0.34 for the left limb and 0.31 for the right limb, reflecting enhanced neuromotor adaptability under cognitive load. Comparatively, the NCI group showed no significant changes in dual-task performance, with *p*-values greater than 0.05 and effect sizes remaining small at 0.16 or less. Post-intervention between-group analysis revealed that initial disparities in gait complexity were mitigated, as no significant differences were found between the CI and NCI groups in either limb.

### 3.2. Longitudinal Analysis of Gait Entropy

**Figure 2:**
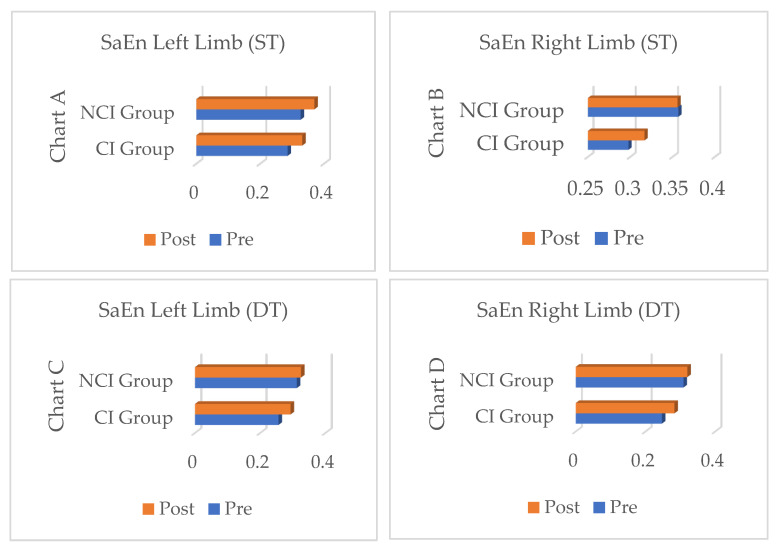
Comparison of Sample Entropy (SaEn) values between left and right lower limbs under Single-Task (ST) and Dual-Task (DT) gait conditions at pre- and post-intervention time points for the Cognitive Impairment (CI) and Control (NCI) groups. Each plot displays the mean SaEn, with connecting lines illustrating the longitudinal change from baseline to post-intervention.

Figure 2 shows the changes in Sample Entropy values over time. In most assessments, both groups showed increases from pre- to post-intervention, indicating a general improvement in movement complexity and neuromotor adaptability.

In the single-task condition for the left limb, the CI group showed a significant increase in Sample Entropy after the intervention. The NCI group showed a small, non-significant increase but maintained higher overall values than the CI group at both time points. In the single-task condition for the right limb, the CI group showed an upward trend, while the NCI group remained stable. In the single-task condition for the right limb, the CI group showed an upward trend, while the NCI group remained stable.

Under the dual-task condition with the left limb, the CI Group exhibited a notable, statistically significant increase in Sample Entropy post-intervention, whereas the NCI Group showed only marginal improvement. Finally, under the dual-task condition for the right limb, both groups showed increases in Sample Entropy, with the increase being significantly greater in the CI Group. The NCI Group started from and maintained a higher baseline level.

## 4. Discussion

This study suggests that an eight-week multicomponent exercise program was associated with improved gait complexity in institutionalized older adults with cognitive impairment. Using a non-linear measure—Sample Entropy—allowed us to detect meaningful changes in neuromotor function that traditional linear measures may not capture [5,15,16,23,33]. These results suggest that structured motor-cognitive training may help reduce mobility decline, especially during cognitively demanding dual-task walking [17,28].

Participants with cognitive impairment showed notable post-intervention increases in Sample Entropy values. While improvements were statistically significant in the left limb across conditions, the right limb under single-task conditions showed a positive trend that did not reach statistical significance, with a small effect size. This distinction is important when compared to the moderate gains observed under dual-task conditions.

Participants with cognitive impairment showed clear increases in Sample Entropy after the intervention. Improvements were statistically significant in the left limb under all conditions. In the right limb during single-task walking, there was a positive trend, but it did not reach statistical significance and showed a small effect size. In contrast, moderate improvements were observed under dual-task conditions.

These bilateral improvements during dual-task walking may reflect better neuromotor adaptability and less rigid gait control under high cognitive load [14,18,34]. The improvements in the left limb during dual-task walking are consistent with previous findings suggesting that motor asymmetry may be reduced in at-risk populations [33,35]. Overall, the findings support the idea that gait problems in cognitive impairment are related to disrupted motor-cognitive integration rather than only peripheral motor decline [36]. The exercise program was associated with meaningful improvements, particularly under dual-task conditions [37].

From a neurobiological perspective, the increase in gait complexity may reflect improved efficiency and flexibility in motor-cognitive control networks involving prefrontal, frontoparietal, and subcortical regions [38]. Repeated combined motor and cognitive training may strengthen coordination across these networks, allowing more adaptive control of stride patterns. Although brain activity was not directly measured, the entropy changes are consistent with neuroplastic adaptations reported in neuroimaging studies.

Non-linear gait measures proved sensitive to changes that traditional linear measures may miss [39]. These findings support the use of entropy-based gait analysis and motor-cognitive exercise in geriatric care, while acknowledging limitations such as study design, sample size, and short follow-up duration.

In contrast, the cognitively healthy group showed only small changes and maintained higher entropy values at both time points, which is consistent with stable aging patterns [5,40]. This suggests that structured interventions may be especially beneficial for individuals with cognitive decline and may promote neuroplastic adaptations [41,42].

The increase in Sample Entropy in participants with cognitive impairment may indicate a reorganization of motor control strategies. This could involve more efficient activation of the prefrontal cortex, which plays a key role in attention during walking [43]. However, this explanation remains hypothetical because brain activity was not directly measured. Improvements under dual-task conditions may also reflect a partial shift away from the “posture-first” strategy, where frail older adults prioritize balance over cognitive performance [8]. These findings align with recent meta-analyses showing that dual-task interventions can improve both cognitive and motor function in mild cognitive impairment [18].

These results also have public health implications and align with global efforts to promote active aging and independence [1,44]. Multicomponent training may help address Motoric Cognitive Risk syndrome through central sensorimotor adaptations [41,45,46,47]. In addition to physical benefits, such programs may improve psychological well-being by providing structure and cognitive stimulation. Group-based and socially enriched environments may further enhance these benefits through increased social engagement [48,49,50,51].

A major strength of this study was the use of wearable sensors and entropy-based gait analysis, which allowed detection of subtle changes in gait dynamics [14,23,24,25]. Future clinical practice may benefit from combining standard functional tests with instrumental assessments such as inertial sensors to improve diagnostic sensitivity [52,53].

The intervention was feasible in an institutional setting. Participants performed dual-task activities such as arithmetic and naming exercises while walking, which may enhance cortical activation and attentional flexibility [54,55]. Improvements under cognitively demanding conditions are functionally meaningful and may support greater independence. These programs provide a non-pharmacological strategy to manage functional decline in dementia and improve quality of life [56,57,58,59].

Increased gait entropy in the cognitive impairment group supports previous findings that motor-cognitive training increases movement variability [17,35]. However, falls were not directly measured, so conclusions about fall risk reduction are based on gait variability changes rather than direct evidence.

### Study Limitations

Several limitations should be considered when interpreting these results. First, the sample size was relatively small (42 participants), which may have reduced the statistical power to detect significant differences in all variables. For example, in the single-task condition for the right limb, a small effect size was observed, but the result was not statistically significant. This may indicate a Type II error, meaning the study may not have had enough power to confirm a real effect. Second, the study used a quasi-experimental design and did not include a randomized non-exercising control group with cognitive impairment. Although the stability of the healthy control group supports internal validity for test–retest effects, the absence of a passive impaired control group makes it difficult to fully separate the effects of the intervention from other environmental or time-related factors. Third, the intervention lasted eight weeks. While this duration was enough to produce initial changes in Sample Entropy, it does not allow conclusions about long-term effects. Because no follow-up assessment was conducted, it is unclear whether the improvements in gait were maintained after the intervention ended.

In addition, cognitive impairment was identified using validated screening tools, but the study did not systematically control for dementia subtype, psychotropic medication use, or the presence of sarcopenia. These factors may affect gait variability and neuromotor control, and not accounting for them limits more detailed interpretation of the mechanisms involved.

Participants were recruited only from institutionalized settings, representing a specific level of frailty. Therefore, the results may not apply to community-dwelling older adults. Future studies should test this protocol in different settings and include kinematic or neuroimaging analyses to better understand the biomechanical and neural mechanisms behind changes in gait entropy.

Finally, the average number of Inter-Stride Intervals used to calculate Sample Entropy was relatively small due to the short walking distance and physical limitations of institutionalized older adults. Although the number of intervals was consistent across groups and time points, shorter time series may reduce the stability of non-linear measurements. Therefore, the entropy results should be interpreted with caution, and future studies should consider longer walking protocols to improve the reliability of complexity analyses.

## 5. Conclusions

This study suggests that an eight-week motor-cognitive exercise program may improve gait complexity in institutionalized older adults with cognitive impairment. Using Sample Entropy allowed the detection of positive neuromotor changes, shown by greater variability and adaptability in gait patterns after the intervention.

Improvements in gait complexity were observed during both single-task and dual-task walking. The consistent gains under cognitive load suggest that the multicomponent program may enhance coordination between motor and cognitive systems. This may help participants manage real-world walking challenges more effectively.

The intervention appears to reduce the motor rigidity associated with cognitive decline and brings the performance of participants with cognitive impairment closer to the baseline levels of cognitively healthy older adults. In addition, non-linear measures proved to be sensitive tools for identifying subtle changes in gait that traditional linear methods may not detect.

These findings support the use of structured and supervised exercise as a non-pharmacological approach to promote independence and improve quality of life in pathological aging. However, because falls were not directly measured, conclusions about reduced fall risk are based on improvements in gait adaptability rather than direct evidence.

### Practical Applications

From a clinical perspective, these findings support the integration of structured motor-cognitive training into standard geriatric care, especially in long-term care facilities. The results show that combining physical exercise with cognitive tasks can help reduce gait rigidity in older adults with cognitive impairment.

Clinicians and healthcare professionals should prioritize dual-task exercises, such as walking while performing cognitive tasks like arithmetic or memory recall, because these activities appear to produce consistent neuromotor improvements. Compared to physical training alone, this multicomponent approach may better prepare individuals for the complex walking demands of daily life.

This study also highlights the importance of using wearable technology and non-linear analysis in routine clinical assessments. Traditional linear measures may miss subtle changes in neuromotor control. Therefore, inertial sensors and measures such as Sample Entropy are recommended for more sensitive monitoring. This method also allows exercise programs to be tailored to each individual’s cognitive and motor abilities.

The high adherence rates and positive outcomes suggest that group-based interventions are a cost-effective, non-pharmacological strategy to promote active aging and maintain functional independence in vulnerable populations.

## Figures and Tables

**Table 1:** Summary of statistical comparisons, descriptive statistics, and effect sizes for Sample Entropy (SaEn).

Comparison/Condition	Descriptive 1 (Mean ± SD; Median)	Descriptive 2 (Mean ± SD; Median)	Test	Statistic	*p*-Value	Sig?	Rosenthal’s *R*	Effect Magnitude
**Between-Group** **(CI vs. NCI)**	**Group CI**	**Group NCI**	**(U)**					
ST—SaEn_1 Pre (Left)	0.286 ± 0.101; 0.279	0.327 ± 0.106; 0.301	Mann–Whitney	135	0.146	No	0.23	Small
ST—SaEn_2 Pre (Right)	0.298 ± 0.097; 0.292	0.357 ± 0.093; 0.348	Mann–Whitney	112	**0.036**	**Yes**	**0.33**	**Moderate**
DT—SaEn_1 Pre (Left)	0.256 ± 0.083; 0.260	0.312 ± 0.067; 0.322	Mann–Whitney	131	0.118	No	0.25	Small
DT—SaEn_2 Pre (Right)	0.247 ± 0.082; 0.257	0.309 ± 0.065; 0.323	Mann–Whitney	130	0.111	No	0.25	Small
**Within-Group (CI)**	**Pre-Intervention**	**Post-Intervention**	**(W)**					
ST—SaEn_1 (Left)	0.286 ± 0.101; 0.279	0.332 ± 0.097; 0.329	Wilcoxon	63	**0.006**	**Yes**	**0.39**	**Moderate**
ST—SaEn_2 (Right)	0.298 ± 0.097; 0.292	0.317 ± 0.083; 0.305	Wilcoxon	116	0.220	No	0.17	Small
DT—SaEn_1 (Left)	0.256 ± 0.084; 0.227	0.293 ± 0.083; 0.260	Wilcoxon	73	**0.015**	**Yes**	**0.34**	**Moderate**
DT—SaEn_2 (Right)	0.247 ± 0.076; 0.215	0.283 ± 0.082; 0.257	Wilcoxon	82	**0.030**	**Yes**	**0.31**	**Moderate**
**Within-Group (NCI)**	**Pre-Intervention**	**Post-Intervention**	**(W)**					
ST—SaEn_1 (Left)	0.327 ± 0.106; 0.301	0.370 ± 0.076; 0.386	Wilcoxon	41	0.303	No	0.19	Small
ST—SaEn_2 (Right)	0.357 ± 0.093; 0.348	0.356 ± 0.063; 0.330	Wilcoxon	67	0.720	No	0.07	Negligible
DT—SaEn_1 (Left)	0.312 ± 0.081; 0.333	0.325 ± 0.067; 0.322	Wilcoxon	47	0.489	No	0.13	Small
DT—SaEn_2 (Right)	0.309 ± 0.080; 0.331	0.320 ± 0.065; 0.323	Wilcoxon	44	0.389	No	0.16	Small

Legend: CI = Cognitive Impairment; NCI = No Cognitive Impairment (Control); ST = Single-Task; DT = Dual-Task; SaEn = Sample Entropy. Effect Magnitude classification based on Cohen (1988) [32].

## Data Availability

The datasets presented in this article are not readily available because the data are part of an ongoing study. Requests to access the datasets should be directed to the corresponding authors.
